# Supplementary material for: The effect of climate change on the distribution of a tropical zoanthid (Palythoa caribaeorum) and its ecological implications
Source: PeerJ. 2018 May 17;6:e4777. doi: 10.7717/peerj.4777 (PMC5960586; doi:10.7717/peerj.4777)
Supplement: Supplemental Information 1 — Files contain all datasets in .csv format, along with the codes used for variable selection in R, and references list with original data. [file peerj-06-4777-s001.zip › Reference list_Palythoa_caribaeorum.docx]

STUDIES AND DATASETS WHERE OCCURRENCE POINTS OF *P. CARIBAEORUM* WERE EXTRACTED FROM

Acosta, A. (2001). Disease in Zoanthids: Dynamics in space and time. Hydrobiologia, 460, 113–130. http://doi.org/10.1023/A:1013135702430

Acosta, A., & González, A. M. (2007). Fission in the Zoantharia Palythoa caribaeorum (Duchassaing and Michelotii, 1860) populations: a latitudinal comparisson. Boletín de Investigaciones Marinas Y Costeras - INVEMAR, 36(1), 151–165.

Almeida, J. G. L., Maia, A. I. V, Wilke, D. V., Silveira, E. R., Braz-Filho, R., La Clair, J. J., … Pessoa, O. D. L. (2012). Palyosulfonoceramides A and B: Unique sulfonylated ceramides from the Brazilian zoanthids Palythoa caribaeorum and Protopalyhtoa variabilis. Marine Drugs, 10(12), 2846–2860. http://doi.org/10.3390/md10122846

Amaral, F. D., Hudson, M. M., Silveira, F. L., Migotto, A. E., Pinto, S. M., & Longo, L. (2000). Cnidarians of Saint Peter and St . Paul Archipelago , Northeast Brazil. In Atlantic (Vol. 9, pp. 567–572).

Amaral, F. M. D., Ramos, C. A. C., Leão, Z. M. A. N., Kikuchi, R. K. P., Lima, K. K. M., Longo, L. L., … Vasconcelos, S. L. (2009). Checklist and morphometry of benthic cnidarians from the Fernando de Noronha Archipelago, Brazil. Cahiers de Biologie Marine, 50(3), 277–290.

Azevedo, C. A. A., Carneiro, M. A. A., Oliveira, S. R., & Marinho-Soriano, E. (2011). Macrolgae as an indicator of the environmental health of the Pirangi reefs, Rio Grande do Norte, Brazil. Revista Brasileira de Farmacognosia, 21(2), 323–328. http://doi.org/10.1590/S0102-695X2011005000071

Bastidas, C., & Bone, D. (1996). Competitive Strategies Between Palythoa Caribaeorum and Zoanthus Sociatus (Cnidaria: Anthozoa) at a Reef Flat Environment in Venezuela. Bulletin of Marine Science, 59(3), 543–555.

Béress, L., Zwick, J., Kolkenbrock, H. J., & Wassermann, O. (1983). A method for the isolation of the Caribbean palytoxin (C-PTX) from the coelenterate (zoanthid). Toxicon, 21(2), 285–290.

Boscolo, H. K., & Silveira, F. L. (2005). Reproductive biology of Palythoa caribaeorum and Protopalythoa variabilis (CNIDARIA, ANTHOZOA, ZOANTHIDEA) from the Southeastern coast of Brazil. Braz. J. Biol, 65(1), 29–41.

Bouzon, J. L., Brandini, F. P., & Rocha, R. M. (2012). Biodiversity of Sessile Fauna on Rocky Shores of Coastal Islands in Santa Catarina, Southern Brazil. Marine Science, 2(5), 39–47. http://doi.org/10.5923/j.ms.20120205.01

Bruce, T., Meirelles, P. M., Garcia, G., Paranhos, R., Rezende, C. E., de Moura, R. L., … Thompson, F. L. (2012). Abrolhos Bank Reef Health Evaluated by Means of Water Quality, Microbial Diversity, Benthic Cover, and Fish Biomass Data. PLoS ONE, 7(6), 13. http://doi.org/10.1371/journal.pone.0036687

Carballeira, N. M., & Reyes, M. (1995). Identification of a new 6-bromo-5,9-eicosadienoic acid from the anemone Condylactis gigantea and the zoanthid Palythoa caribaeorum. Journal of Natural Products, 58(11), 1689–94.

Castro, C. B., Amorim, L. C. de, Calderon, E. N., & Segal, B. (2006). Cobertura e recrutamento de corais recifais (Cnidaria: Scleractinia e Milleporidae) nos recifes Itacolomis, Brasil. Arquivos Do Museu Nacional, 64(1), 29–40.

Castro, C. B. e, Segal, B., Negrão, F., & Calderon, E. N. (2012). Four-year monthly sediment deposition on turbid southwestern Atlantic coral reefs, with a comparison of benthic assemblages. Brazilian Journal of Oceanography, 60(1), 49–63. http://doi.org/10.1590/S1679-87592012000100006

Chimetto, L. A., Brocchi, M., Gondo, M., Thompson, C. C., Gomez-Gil, B., & Thompson, F. L. (2009). Genomic diversity of vibrios associated with the Brazilian coral Mussismilia hispida and its sympatric zoanthids (Palythoa caribaeorum, Palythoa variabilis and Zoanthus solanderi). Journal of Applied Microbiology, 106(6), 1818–1826. http://doi.org/10.1111/j.1365-2672.2009.04149.x

Chimetto, L. A., Brocchi, M., Thompson, C. C., Martins, R. C. R., Ramos, H. R., & Thompson, F. L. (2008). Vibrios dominate as culturable nitrogen-fixing bacteria of the Brazilian coral Mussismilia hispida. Systematic and Applied Microbiology, 31(4), 312–319. http://doi.org/10.1016/j.syapm.2008.06.001

Colvard, N. B., & Edmunds, P. J. (2011). Decadal-scale changes in abundance of non-scleractinian invertebrates on a Caribbean coral reef. Journal of Experimental Marine Biology and Ecology, 397(2), 153–160. http://doi.org/10.1016/j.jembe.2010.11.015

Correia, M. D., & Sovierzoski, H. H. (2008). Macrobenthic diversity reaction to human impacts on Maceio coral reefs, Alagoas, Brazil. In 11th International Coral Reef Symposium (pp. 7–11). Ft. Lauderdale, Florida.

Costa, D. L., Gomes, P. B., Santos, A. M., Valenca, N. S., Vieira, N. a, & Perez, C. D. (2011). Morphological plasticity in the reef zoanthid Palythoa caribaeorum as an adaptive strategy. Annales Zoologici Fennici, 48(6), 349–358. http://doi.org/10.5735/086.048.0602

Creed, J., Pires, D., & Figueiredo, M. (2007). Biodiversidade marinha da Baía da Ilha Grande. (M. do M. A. – MMA, Ed.)BIODIVERSIDADE 23. Braília, Esplanada dos Ministérios.

Cruz, I. C. S., de Kikuchi, R. K. P., Longo, L. L., & Creed, J. C. (2015). Evidence of a phase shift to Epizoanthus gabrieli Carlgreen, 1951 (Order Zoanthidea) and loss of coral cover on reefs in the Southwest Atlantic. Marine Ecology, 36(3), 318–325. http://doi.org/10.1111/maec.12141

Cruz, I. C. S., Kikuchi, R. K. P., & Leão, Z. M. A. N. (2008). Use of the video transect method for characterizing the Itacolomis reefs, eastern Brazil. Brazilian Journal of Oceanography, 56(4), 271–280. http://doi.org/10.1590/S1679-87592008000400002

Cruz, I. C. S., Kikuchi, R. K. P., & Leão, Z. M. A. N. (2009). Characterization of Coral Reefs from Todos os Santos Bay Protected Area for Management Purpose , Bahia , Brazil. Journal of Integrated Coastal Zone Management, 9(3), 3–23.

Cubit, J., & Williams, S. (1983). The invertebrates of Galeta Reef (caribbean Panama): a species list and bibliography. Atoll Research Bulletin, 269, 44.

Duchassaing, P., & Michelotti, G. (1860). Mémoire sur les coralliaires des antilles. Imprimerie Royale, 256.

Echeverría, C. A., Pires, D. O., Medeiros, M. S., & B, C. C. (1997). Cnidarians od the Atol das Rocas, Brazil. Proc. 8th Int. Coral Reef Sym., (1), 443–446. http://doi.org/10.1007/s13398-014-0173-7.2

Edmunds, P. J. (2000). Patterns in the distribution of juvenile corals and coral reef community structure in St. John, US Virgin Islands. Mar Ecol Prog Ser, 202, 113–124.

Edwards, A., & Lubbock, R. (1983). The ecology of Saint Paul’s Rocks (Equatorial Atlantic). Journal of Zoology, 200(1), 51–69. http://doi.org/10.1111/j.1469-7998.1983.tb06108.x

Fadlallah, Y. H., Karlson, R. H., & Sebens, K. P. (1984). A comparative study of sexual reproduction in three species of panamanian zoanthids (coelenterata: anthozoa). Bulletin of Marine Science, 35(1), 80–89.

Fautin, D. G. (2013). Hexacorallians of the World.

Feitosa, J. L. L., & Ferreira, B. P. (2015). Distribution and feeding patterns of juvenile parrotfish on algal-dominated coral reefs. Marine Ecology, 36(3), 462–474. http://doi.org/10.1111/maec.12154

Francini-Filho, R. B., Coni, E. O. C., Meirelles, P. M., Amado-Filho, G. M., Thompson, F. L., Pereira-Filho, G. H., … Moura, R. L. (2013). Dynamics of Coral Reef Benthic Assemblages of the Abrolhos Bank, Eastern Brazil: Inferences on Natural and Anthropogenic Drivers. PLoS ONE, 8(1), 1–12. http://doi.org/10.1371/journal.pone.0054260

Francini-Filho, R. B., & Moura, R. L. de. (2010). Predation on the toxic zoanthid Palythoa caribaeorum by reef fishes in the abrolhos bank, eastern Brazil. Brazilian Journal of Oceanography, 58(1), 77–79. http://doi.org/10.1590/S1679-87592010000100008

Gleibs, S., & Mebs, D. (1999). Distribution and sequestration of palytoxin in coral reef animals. Toxicon, 37(11), 1521–7.

Gobierno de Miranda. (n.d.). Biodiversidad Marina del Estado Miranda. Retrieved from http://biodiversidadmiranda.cbm.usb.ve/cms/

Haywick, D. W., & Mueller, E. M. (1997). Sediment retention in encrusting Palythoa spp. - A biological twist to a geological process. Coral Reefs, 16(1), 39–46. http://doi.org/10.1007/s003380050057

Hines, D. E., & Pawlik, J. R. (2012). Assessing the antipredatory defensive strategies of Caribbean non-scleractinian zoantharians (Cnidaria): is the sting the only thing? Marine Biology, 159, 389–398. http://doi.org/10.1007/s00227-011-1816-2

Karlson, R. H. (1980). Alternative competitive strategies in a periodically disturbed habitat. Bulletin of Marine Science, 30(4), 894–900.

Kemp, D. W., Cook, C. B., LaJeunesse, T. C., & Brooks, W. R. (2006). A comparison of the thermal bleaching responses of the zoanthid Palythoa caribaeorum from three geographically different regions in south Florida. Journal of Experimental Marine Biology and Ecology, 335(2), 266–276. http://doi.org/10.1016/j.jembe.2006.03.017

Koehl, M. A. R. (1977). Water flow and the morphology of zoanthid colonies. In Third International Coral Reef Symposium (pp. 437–444).

Lajeunesse, T. C. (2002). Diversity and community structure of symbiotic dinoflagellates from Caribbean coral reefs. Marine Biology, 141, 387–400. http://doi.org/10.1007/s00227-002-0829-2

Lesser, M. P., Stochaj, W. R., Tapley, D. W., & Shick, J. M. (1990). Bleaching in coral reef anthozoans: effects of irradiance, ultraviolet radiation, and temperature on the activities of protective enzymes against active oxygen. Coral Reefs, 8(4), 225–232. http://doi.org/10.1007/BF00265015

Loiola, M., Cruz, I. C. S., Leão, Z. M. A. N., & Kikuchi, R. K. P. (2014). Definition of priority areas for the conservation of a coastal reef complex in the eastern Brazilian coast. Journal of Integrated Coastal Zone Management, 14(4), 611–624. http://doi.org/10.5894/rgci449

Longo, G. O., Krajewski, J. P., Segal, B., & Floeter, S. R. (2012). First record of predation on reproductive Palythoa caribaeorum (Anthozoa: Sphenopidae): insights on the trade-off between chemical defences and nutritional value. Marine Biodiversity Records, 5(e29), 3. http://doi.org/10.1017/S1755267212000206

Maccord, F. S., & Duarte, L. F. L. (2002). Dispersion in Populations of Tropiometra carinata (Crinoidea: Comatulida) in the Sã o Sebastiã o Channel, Sã o Paulo State, Brazil. Estuarine, Coastal and Shelf Science, 54, 219–225. http://doi.org/10.1006

Mantelatto, M. C. (2012). Distribuição e abundância do coral invasor Tubastraea spp. Universidade do Estado do Rio de Janeiro.

Marino, A. (2003). Compilation of marine biota data from Master and PhD studies in Brazil. Retrieved from http://www.vliz.be/en/imis?module=dataset&dasid=4875

Matthews-Cascon, H., & Lotufo, T. M. da. (2006). Biota Marinha da Costa Oeste do Ceará. Brasília: MMA.

Medeiros, P. R., Grempel, R. G., Souza, A. T., Ilarri, M. I., & Rosa, R. S. (2010). Non-random reef use by fishes at two dominant zones in a tropical, algal-dominated coastal reef. Environmental Biology of Fishes, 87(3), 237–246. http://doi.org/10.1007/s10641-010-9593-1

Melo, L. F. de A., Camara, C. A. G. da, Oliveira, L. L. D. da S. S. de, Modesto, J. C. de A., & Pérez, C. D. (2012). Toxicity against Artemia salina of the zoanthid Palythoa caribaeorum (Cnidaria: Anthozoa) used in folk medicine on the coast of Pernambuco, Brazil. Biotemas, 25(3), 145–151. http://doi.org/10.5007/2175-7925.2012v25n3p145

Mendonça-Neto, J. P., Ferreira, C. E. L., Chaves, L. C. T., & Pereira, R. C. (2008). Influence of Palythoa caribaeorum (Anthozoa, Cnidaria) zonation on site-attached reef fishes. Anais Da Academia Brasileira de Ciencias, 80(3), 494–513. http://doi.org/10.1590/S0001-37652008000300010

Oigman-Pszczol, S. S., Figueiredo, M. A. de O., & Creed, J. C. (2004). Distribution of Benthic Communities on the Tropical Rocky Subtidal of Armacao dos Buzios, Southeastern Brazil. Marine Ecology, 25(3), 173–190. http://doi.org/10.1111/j.1439-0485.2004.00018.x

Pereira, A. P. V. (2007). Caracterização Fisionômica da Comunidade Marinha Bentônica de Substrato Consolidado do Infralitoral no Costão Oeste da Enseada das Palmas, Parque Estadual da Ilha Anchieta, Ubatuba - SP, Brasil.

Pérez, C. D., Vila-Nova, D. a., & Santos, a. M. (2005). Associated community with the zoanthid Palythoa caribaeorum (Duchassaing & Michelotti, 1860) (Cnidaria, Anthozoa) from littoral of Pernambuco, Brazil. Hydrobiologia, 548(1), 207–215. http://doi.org/10.1007/s10750-005-5441-2

Rabelo, E. F. (2007). Distribuição espacial e interações competitivas em zoantídeos (cnidária: zoanthidae) em um ambiente de recifes de arenito no nordeste do Brasil. Universidade Federal do Ceará.

Ramos, C. A. C., Amaral, F. D., de Kikuchi, R. K. P., Chaves, E. M., & de Melo, G. R. (2010). Quantification of reef benthos communities and variability inherent to the monitoring using video transect method. Environmental Monitoring and Assessment, 162(1–4), 95–101. http://doi.org/10.1007/s10661-009-0778-z

Robertson, R. (1967). Heliacus (Gastropoda: Architectonicidae) symbiotic with Zoanthiniaria (Coelenterata). Science (New York, N.Y.), 156(3772), 246–8.

Sebens, K. (1977). Autotrophic and heterotrophic nutrition of coral reef zoanthids. In Proceedings of the 3rd International Coral Reef Symposium (Vol. 1, pp. 397–404).

Sebens, K. P. (1982). Intertidal distribuition of Zoanthids on the Caribbean Coast of Panamá: Effects of predation and desiccation. Bulletin of Marine Science, 32(1), 316–335.

Seemann, P., Gernert, C., Schmitt, S., Mebs, D., & Hentschel, U. (2009). Detection of hemolytic bacteria from Palythoa caribaeorum (Cnidaria, Zoantharia) using a novel palytoxin-screening assay. Antonie van Leeuwenhoek, 96(4), 405–11. http://doi.org/10.1007/s10482-009-9353-4

Segal, B., & Castro, C. B. (2011). Coral community structure and sedimentation at different distances from the coast of the Abrolhos Bank, Brazil. Brazilian Journal of Oceanography, 59(2), 119–129. http://doi.org/10.1590/S1679-87592011000200001

Silveira, Fabio Lang da Lopes, R. M. (2008). Marine Biodiversity in Ilha Grande Bay, Rio de Janeiro State, Southwest Brazil. Retrieved from http://idn.ceos.org/portals/Metadata.do?Portal=idn_ceos&KeywordPath=%5BParameters%3A+Topic%3D%27BIOLOGICAL+CLASSIFICATION%27%2C+Term%3D%27ANIMALS%2FINVERTEBRATES%27%2C+Variable_Level_1%3D%27CNIDARIANS%27%2C+Variable_Level_2%3D%27HYDROZOANS%27%5D&OrigMetad

Silveira, F. L. Da, & Morandini, A. C. (2011). Checklist dos Cnidaria do Estado de São Paulo, Brasil. Biota Neotrop., 11(111a).

Soares, C. L. S., Pérez, C. D., Maia, M. B. S., Silva, R. S., & Melo, L. F. A. (2006). Avaliação da atividade antiinflamatória e analgésica do extrato bruto hidroalcoólico do zoantídeo Palythoa caribaeorum (Duchassaing &amp; Michelotti, 1860). Revista Brasileira de Farmacognosia, 16(4), 463–468. http://doi.org/10.1590/S0102-695X2006000400004

Soares, M. D. O., Rabelo, E. F., & Mathews-cascon, H. (2011). Intertidal Anthozoans from the coast of Ceará ( Brazil ). Brazilian Journal of Biosciences, 9(4), 437–443.

Souza, D. S. L., Grossi-de-Sa, M. F., Silva, L. P., Franco, O. L., Gomes-Junior, J. E., Oliveira, G. R., … Abreu, L. R. D. (2008). Identification of a novel beta-N-acetylhexosaminidase (Pcb-NAHA1) from marine Zoanthid Palythoa caribaeorum (Cnidaria, Anthozoa, Zoanthidea). Protein Expression and Purification, 58(1), 61–9. http://doi.org/10.1016/j.pep.2007.10.024

Stampar, S. N., Silva, P. F. da, & Osmar, J. L. J. (2007). Predation on the Zoanthid Palythoa caribaeorum (Anthozoa, Cnidaria) by a Hawksbill Turtle (Eretmochelys imbricata) in Southeastern Brazil. Marine Turtle Newsletter, (117), 2–5.

Steiner, A. Q., Amaral, F. M. D., Amaral, J. R. de B. C. do, Sassi, R., & Barradas, J. I. (2015). Zonação de recifes emersos da Área de Proteção Ambiental Costa dos Corais, Nordeste do Brasil. Iheringia. Série Zoologia, 105(2), 184–192. http://doi.org/10.1590/1678-476620151052184192

Suchanek, T. H., & Green, D. J. (1981). Interspecific competition between Palythoa caribaeorum and other sessile invertebrates on St. Croix reefs, U.S. Virgin Islands. In Proceedings of the 4th International Coral Reef Symposium (Vol. 2, pp. 679–684). Manila.

Varela, C., Guitart, B., Ortiz, M., & Lalana, R. (2002). Los zoantídeos (cnidaria, anthozoa, zoanthiniaria), de la región occidental de Cuba. Rev. Invest. Mar, 23(3), 179–184.

Villaça, R., & Pitombo, F. B. (1997). Benthic communities of shallow-water reefs of abrolhos, brazil. Brazilian Journal of Oceanography, 45(1/2), 35–43.

Villamizar, E., Camisotti, H., Rodríguez, B., Pérez, J., & Romero, M. (2008). Impacts of the 2005 Caribbean bleaching event at Archipiélago de Los Roques National Park, Venezuela. Revista de Biología Tropical, 56(1), 255–270.
